# Peripheral Blood Gene Expression and Protein Profiles of Purinergic and Glutamatergic Signaling Components and PD-L1 in Gastric Cancer: A Cross-Sectional Study

**DOI:** 10.3390/cimb48070696

**Published:** 2026-07-09

**Authors:** Hakki Coskun, Zuhal Tuncbilek, Husnu Cagri Genc, Gulcihan Cinar Kaya, Ayca Tas

**Affiliations:** 1 Department of General Surgery, Faculty of Medicine, Sivas Cumhuriyet University, 58140 Sivas, Türkiye; cagrigenc@cumhuriyet.edu.tr; 2 Department of Chemistry and Chemical Processing Technologies Services, Yıldızeli Vocational School, Biochemistry Program, Sivas Cumhuriyet University, 58140 Sivas, Türkiye; zuhaltuncbilek@cumhuriyet.edu.tr; 3 Department of Medical Services and Techniques, Yıldızeli Vocational School, Medical Laboratory Techniques Program, Sivas Cumhuriyet University, 58140 Sivas, Türkiye; gulcihancinarkaya@cumhuriyet.edu.tr; 4 Department of Biochemistry, Faculty of Medicine, Sivas Cumhuriyet University, 58140 Sivas, Türkiye; aycatas@cumhuriyet.edu.tr

**Keywords:** gastric cancer, purinergic signaling, glutamatergic Signaling, oxidative stress

## Abstract

Gastric cancer remains a major cause of cancer-related mortality worldwide, highlighting the need for an improved understanding of its molecular mechanisms. Purinergic and glutamatergic signaling pathways, immune checkpoint molecules, and oxidative stress are thought to contribute to tumor biology; however, their combined evaluation in gastric cancer is limited. Methods: Gene expression levels of glutamate receptor ionotropic *N-Methyl-D-aspartate 2A (GRIN2A)*, *purinergic receptor P2X1 (P2RX1)*, and *programmed death-ligand 1 (PD-L1/CD274)* were analyzed using real-time PCR, while serum protein levels were determined by ELISA. Total antioxidant status (TAS) and total oxidant status (TOS) were also measured to assess systemic oxidative balance. Results: Gene expression analysis revealed an increased expression trend for *P2RX1* in the gastric cancer group compared with healthy controls, whereas *GRIN2A* and *CD274 (PD-L1)* expression levels showed numerical decreases. Serum concentrations of GRIN2A and PD-L1, as well as TAS and TOS levels, were numerically higher in patients with gastric cancer; however, none of these differences reached statistical significance (*p* > 0.05). Likewise, serum P2RX1 levels did not differ significantly between groups. Receiver operating characteristic (ROC) analysis demonstrated area under the curve (AUC) values close to 0.5 for all evaluated parameters, indicating limited discriminatory and diagnostic performance. Conclusions: These findings suggest that these molecular markers may reflect disease-related biological alterations rather than serve as independent circulating diagnostic biomarkers in gastric cancer.

## 1. Introduction

Gastric cancer is a major gastrointestinal malignancy characterized by high mortality rates and frequent late-stage diagnoses. According to the latest GLOBOCAN 2022 estimates, approximately 968,784 new cases and 660,175 deaths were reported worldwide, making gastric cancer the fifth most commonly diagnosed cancer and the fifth leading cause of cancer-related mortality globally [1]. It remains particularly prevalent in Eastern Asia, Eastern Europe, and parts of South America, where the disease burden is disproportionately high [2]. The absence of distinct clinical symptoms during the early stages often results in disease progression by the time of diagnosis, thereby adversely affecting both treatment success and overall survival [3]. In addition, increasing evidence suggests that circulating molecular biomarkers obtained from peripheral blood may reflect systemic inflammatory and immunological alterations associated with gastric cancer and may provide minimally invasive tools for disease monitoring and prognostic evaluation [4].

N-Methyl-D-aspartate 2A (GRIN2A), an important member of the glutamatergic receptor family, functions as an N-methyl-D-aspartate receptor (NMDA receptor) subunit that regulates intracellular calcium influx and may contribute to key tumor-related processes, such as proliferation, migration, and chemoresistance [5]. Recent studies have suggested that NMDA receptor signaling may contribute to tumor cell survival, calcium-dependent oncogenic signaling pathways, metabolic adaptation, and resistance to apoptosis in several malignancies [6]. Similarly, P2RX1 regulates ATP-mediated ion fluxes associated with inflammatory signaling, neutrophil activation, platelet aggregation, and immune cell communication within the tumor microenvironment, suggesting a potential role in systemic cancer-associated immune modulation [7]. Although P2RX7 has been extensively investigated in cancer biology, emerging evidence indicates that other purinergic receptors, including P2RX1, may also participate in tumor associated inflammatory signaling and systemic immune responses [8].

PD-L1 is a central component of tumor immune evasion mechanisms, and its increased expression levels are directly associated with tumor aggressiveness and therapeutic response [9]. Furthermore, circulating *CD274* expression profiles detected in the peripheral blood have recently attracted attention as potential indicators of systemic immune status and therapeutic responsiveness in gastrointestinal malignancies [10]. The simultaneous evaluation of alterations in these molecules at both the protein and gene expression levels is of great importance for a more comprehensive understanding of tumor biology and for identifying novel diagnostic and prognostic targets.

GRIN2A and P2RX1 were selected as candidate biomarkers because glutamatergic and purinergic signaling pathways have increasingly been implicated in tumor progression, immune regulation, and interactions within the tumor microenvironment [6,7,8]. PD-L1 was included because it is a well-established immune checkpoint molecule involved in tumor immune evasion and has recognized prognostic and therapeutic relevance in gastric cancer [9,10]. Although GRIN2A and P2RX1 are predominantly membrane-associated receptors, accumulating evidence indicates that membrane-associated proteins can be detected in the circulation through extracellular vesicles, proteolytic ectodomain shedding, apoptotic bodies, and passive release from damaged or dying cells [4]. Consequently, circulating concentrations of these proteins may reflect systemic tumor-associated molecular and immunological alterations rather than direct receptor abundance within tumor tissues [4]. Therefore, GRIN2A, P2RX1, and PD-L1 were selected to investigate whether alterations in glutamatergic signaling, purinergic signaling, and immune checkpoint regulation are reflected in the peripheral circulation of patients with gastric cancer [4,6,7,8,9,10].

Another critical determinant of cancer pathophysiology is oxidative stress, as excessive production of reactive oxygen species can trigger DNA damage, dysregulation of signaling pathways, and immune suppression in tumor cells [11]. Therefore, the assessment of total antioxidant and oxidant statuses provides a complementary approach for understanding the biochemical balance that shapes tumor development. The current literature emphasizes that the interaction between oxidative stress markers, immune checkpoint molecules, and purinergic and glutamatergic receptor systems plays a pivotal role in the progression of gastric cancer [12]. Oxidative stress may also modulate purinergic and glutamatergic signaling pathways through reactive oxygen species-mediated alterations in calcium homeostasis, inflammatory signaling cascades, and immune checkpoint activation, thereby contributing to tumor progression and immune escape mechanisms [13].

In this context, the evaluation of *GRIN2A*, *P2RX1*, and *PD-L1* levels at both the protein and gene expression levels in blood samples obtained from gastric cancer patients and healthy individuals, alongside the simultaneous analysis of oxidative stress parameters, offers a comprehensive approach to elucidate the biomolecular dynamics of the disease. This study was specifically designed to investigate minimally invasive circulating biomarkers that may reflect the systemic molecular and immunological alterations associated with gastric cancer. Peripheral blood based analyses provide a practical and clinically accessible approach for screening, disease monitoring, and longitudinal patient follow up compared with tissue-based procedures. Although tumor tissue biopsies may provide a more direct representation of the tumor microenvironment and tumor specific molecular alterations, blood-based biomarkers may still offer important advantages for early detection strategies and repeated clinical assessments because of their minimally invasive nature. This strategy may enable the identification of novel biomarkers involved in gastric cancer pathogenesis and the determination of potential molecular targets capable of supporting clinical decision-making. Accordingly, this study aimed to comparatively evaluate the protein and gene expression levels of *GRIN2A*, *P2RX1*, and *CD274* in patients with gastric cancer and healthy individuals and to investigate the relationship between alterations in these biomarkers and oxidative stress parameters, thereby identifying potential diagnostic and prognostic markers that may shed light on the pathogenesis of gastric cancer.

## 2. Patients and Methods

### 2.1. Study Population

A total of 90 individuals were enrolled in the present study, including 45 patients with clinically confirmed gastric cancer and 45 age- and sex-matched healthy controls. Patients diagnosed with primary gastric cancer at the Department of General Surgery, Sivas Cumhuriyet University Faculty of Medicine Hospital, were included in the study. Healthy volunteers without a history of malignancy or chronic systemic disease, identified through routine clinical evaluation, were recruited as the control group. Although individuals aged 18 years and older were eligible for inclusion, all participants enrolled during the study period were over 40 years of age, reflecting the age distribution of patients presenting to our center, histopathologically confirmed gastric cancer diagnosis, and an anticipated life expectancy of more than six months. Individuals with severe cardiovascular or neurological disorders, additional malignancies, ischemic diseases, chronic inflammatory disorders, acute or chronic infectious diseases, autoimmune diseases, or conditions associated with immunosuppression were excluded from the study. Peripheral blood samples were collected in the morning following overnight fasting whenever possible, and sample collection from patients was performed prior to chemotherapy or immunotherapy administration. Patients will be randomly selected with no restrictions based on age or gender. Prior to enrollment, written informed consent was obtained from all participants. Using the G. Power-3.1.9.4 software, a power analysis was performed with p(1 − β) = 80% power, α = 0.05 effect size (d) = 0.61, and a group size of 2. A total of 90 individuals, with 45 people in each group, were found to be sufficient. The power of the study was found to be 0.8076051. IBM SPSS Statistics 23.0 program will be used for data analysis. The suitability of numerical variables to normal distribution will be examined with Shapiro-Wilk or Kolmogorov-Smirnov tests. Numerical variables will be presented as mean, standard deviation, and median (min-max). The study protocol was approved by the Sivas Cumhuriyet University Health Sciences Research Ethics Committee (approval date: 30 November 2025; decision no:2025-10/54), and all procedures were conducted in accordance with the ethical principles of the Declaration of Helsinki.

### 2.2. Blood Specimens

Peripheral blood samples were collected in the morning after an overnight fast to minimize the effects of circadian variation and postprandial metabolic changes on circulating biochemical and molecular biomarkers, thereby ensuring standardized sample collection across all participants. Blood samples (3–4 mL) were collected into serum blood collection tubes, centrifuged at 3000 rpm for 10 min within 1 h of collection to obtain serum for subsequent analyses. The separated serum samples were stored at −80 °C until the day of analysis.

### 2.3. RNA Isolation and cDNA Synthesis

Total RNA extraction was carried out from EDTA-treated whole blood samples using a commercially available kit (RNeasy Plus Mini Kit, QIAGEN, Hilden, Germany; Cat. No. 74104). RNA purity and concentration were determined via spectrophotometric assessment of the 260/280 nm ratio. Complementary DNA (cDNA) (A.B.T., Berlin, Germany) synthesis was then performed by reverse transcription, and the synthesized cDNA samples were stored at −20 °C for downstream analyses.

Gene expression was quantified from RNA isolated from peripheral whole blood, whereas protein concentrations were measured in serum. These analyses represent distinct biological compartments and were therefore evaluated independently rather than being interpreted as direct molecular equivalents.

### 2.4. Gene Expression Analysis

Commercially available, pre-designed, and manufacturer-validated optimized primers were used for all target genes. Because these primer sets are proprietary, the exact primer sequences were not publicly disclosed by the manufacturer. Gene expression analysis was performed using commercially available, pre-designed, and manufacturer-validated human primer assays (AXACELL Bioassay, Tokat, Türkiye). The following primer assays were used: *GRIN2A* (PRT-0544-HU), *P2RX1* (PRT-1331-HU), *CD274/PD-L1* (PRT-0712-HU), and *Glyceraldehyde-3-phosphate dehydrogenase (GAPDH)* (PRT-0001-HU). All qRT-PCR reactions were technically performed in duplicate. Amplification specificity was verified by melting curve analysis, and only reactions showing a single specific melting peak were included in the analysis. PCR efficiency was evaluated according to the manufacturer’s recommendations and found to be within the acceptable range for relative quantification. Analysis was carried out using the Eva Green Master Mix (Cat. No: 60R-01-100, SNP Biyoteknoloji, Ankara, Türkiye) according to the manufacturer’s instructions. The analysis was performed using a Rotor-Gene 6000 RT-PCR system (QIAGEN, Germany). GAPDH was used as the internal control (housekeeping) gene for normalization of gene expression levels. Cycle threshold (Ct) values were normalized, and relative gene expression levels were calculated using the 2^−ΔΔCt^ method.

### 2.5. Measurement of Serum GRIN2A, P2RX1, and PD-L1 Protein Levels

Serum protein levels of GRIN2A (Cat. No.:E4452Hu, BT LAB, Jiaxing, China), P2RX1 (Cat. No.: E5824Hu, BT LAB, Jiaxing, China), and PD-L1 (Cat. No.:E2153Hu, BT LAB, Jiaxing, China) were measured using ELISA kits following the assay protocol provided. According to the manufacturers’ specifications, the ELISA kits used in the study had standard curve ranges of 10–2000 ng/L for GRIN2A (sensitivity: 5.24 ng/L), 0.1–40 ng/mL for P2RX1 (sensitivity: 0.051 ng/mL), and 10–2000 ng/L for PD-L1 (sensitivity: 5.58 ng/L). For all protein measurements, intra-assay variability remained below 8%, while inter-assay variability did not exceed 10%. All ELISA measurements were performed according to the manufacturer’s instructions. Serum samples, standards, and quality controls were analyzed in duplicate, and the mean absorbance value was used for statistical analysis. Standard curves were generated for each assay, and samples with absorbance values outside the linear range of the standard curve were excluded or reanalyzed when necessary. Intra-assay and inter-assay coefficients of variation were maintained within the limits specified by the manufacturer. All absorbance values were measured spectrophotometrically at a wavelength of 450 nm.

### 2.6. Determination of Total Antioxidant and Oxidant Status

Total antioxidant status (TAS) and total oxidant status (TOS) levels were measured spectrophotometrically in serum samples using commercially available assay kits (Rel Assay Diagnostics, Mega Tip, Gaziantep, Turkey) according to the manufacturer’s instructions. TOS values were expressed as μmol H_2_O_2_ equivalent/L, and TAS values were expressed as mmol Trolox equivalent/L.

### 2.7. Survival Analysis

To further evaluate the prognostic significance of the investigated biomarkers, Kaplan–Meier survival analyses were performed using the publicly available Kaplan–Meier Plotter database (https://kmplot.com/analysis/), (accessed on 28 June 2026).which integrates gene expression and clinical outcome data from The Cancer Genome Atlas (TCGA) and Gene Expression Omnibus (GEO) datasets for gastric cancer. Overall survival (OS) was analyzed for *GRIN2A* (206534_at), *P2RX1* (210401_at), and *CD274* (227458_at). Patients were automatically stratified into high- and low-expression groups using the optimal cut-off value determined by the Kaplan–Meier Plotter algorithm. Hazard ratios (HRs), 95% confidence intervals (95% CIs), log-rank *p* values, and median survival times were obtained directly from the database. A two-sided *p* value < 0.05 was considered statistically significant.

### 2.8. Statistical Analysis

Statistical analyses were performed using IBM SPSS Statistics version 23.0 (IBM Corp., Armonk, NY, USA). The distribution characteristics of continuous variables were evaluated using the Shapiro–Wilk normality test. Since most of the continuous variables did not meet the assumption of normal distribution, descriptive statistics were presented as median values with minimum and maximum ranges. Categorical variables, such as gender, were expressed as number and percentage. Comparisons between the gastric cancer and healthy control groups were performed according to the distribution pattern of the data. For continuous variables with non-normal distribution, the Mann–Whitney U test was used to compare differences between the two independent groups. Categorical variables were compared using the chi-square test where appropriate. The diagnostic performance of GRIN2A, P2RX1, PD-L1, TAS, and TOS parameters in distinguishing gastric cancer patients from healthy controls was evaluated using receiver operating characteristic (ROC) curve analysis. Area under the curve (AUC), 95% confidence interval (CI), cut-off values, sensitivity, and specificity were calculated for each parameter. Multivariable binary logistic regression analysis was performed to assess the independent associations of serum GRIN2A, P2RX1, PD-L1, TAS, and TOS levels with gastric cancer status. All biomarkers were entered simultaneously into the model using the enter method. Gastric cancer status was coded as 1 and healthy control status as 0. Odds ratios (ORs) with 95% confidence intervals (CIs) were calculated. Spearman’s rank correlation analysis was performed to assess associations among demographic variables, serum biomarker levels, RT-qPCR Ct values, and oxidative stress parameters. Correlation analyses were conducted in the overall study population and separately within the gastric cancer and healthy control groups. Spearman’s correlation coefficients (*r*) and corresponding *p*-values were reported. A *p*-value of <0.05 was considered statistically significant.

## 3. Results

### 3.1. Demographic and Clinical Characteristics

A total of 45 patients with gastric cancer and 45 healthy controls were included in the study. Female participants accounted for 24.4% of the gastric cancer group and 26.7% of the healthy control group, whereas males accounted for 75.6% and 73.3%, respectively. Gender distribution did not differ significantly between the groups (OR = 0.89, 95% CI: 0.35–2.30; *p* = 0.809). The mean age was 63.93 ± 9.91 years in the gastric cancer group and 65.64 ± 10.89 years in the healthy control group. Bootstrap 95% confidence intervals for the mean age were 61.04–66.62 and 62.25–68.82 years, respectively. There was no statistically significant difference in age between the groups (*p* = 0.438; Hedges’ g = −0.16, 95% CI: −0.57 to 0.25). The age ranges were 40–84 years in the gastric cancer group and 45–87 years in the healthy control group. Although the inclusion criteria allowed individuals aged 18 years and older, all participants enrolled in the present study were over 40 years of age (Table 1).

### 3.2. Evaluation of GRIN2A, P2RX1, and PD-L1 Gene Expression Levels

**Figure 1:**
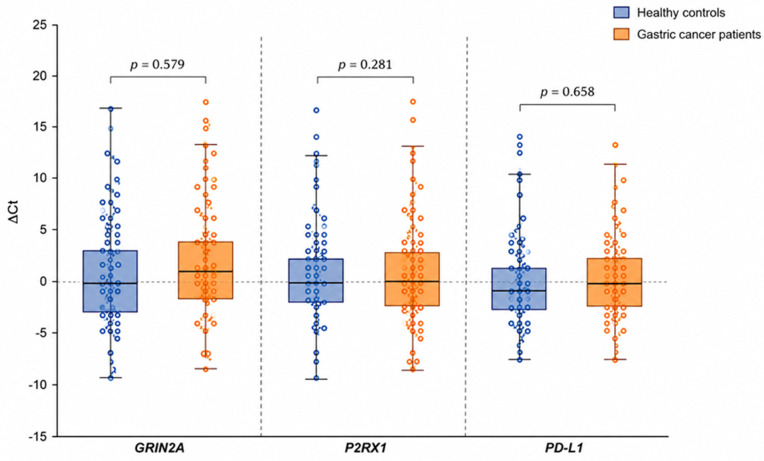
Distribution of Δ_Ct_ values for *GRIN2A*, *P2RX1*, and *PD-L1* in GC patients and healthy controls. Box plots represent the median and interquartile range; whiskers indicate the minimum and maximum values. Individual data points are overlaid. *p*-values were calculated using the Mann–Whitney U test.

Gene expression analysis was performed using RT-PCR following cDNA synthesis from RNA obtained from blood samples collected from individuals in the study groups. The expression levels of *GRIN2A*, *P2RX1*, and *PD-L1* were evaluated using ΔCt and ΔΔCt values, with *GAPDH* used as the reference gene. *P2RX1* demonstrated a numerically higher relative fold-change in the gastric cancer group compared with the control group (2.19-fold). In contrast, *PD-L1* and *GRIN2A* demonstrated numerically lower relative fold-change values (0.56- and 0.43-fold, respectively). However, none of the observed differences in gene expression reached statistical significance (all *p* > 0.05) (Table 2, Figure 1). Therefore, these findings should be interpreted as exploratory numerical patterns rather than evidence of differential gene expression between the groups.

### 3.3. Comparison of GRIN2A, P2RX1, and PD-L1 Protein Levels Between Study Groups

**Figure 2:**
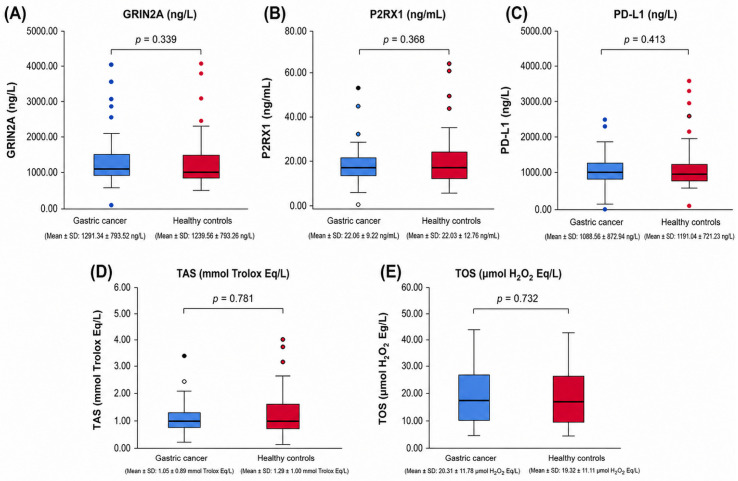
Serum levels of (**A**) GRIN2A, (**B**) P2RX1, (**C**) PD-L1, (**D**) TAS, and (**E**) TOS in patients with GC and healthy controls. Box plots show the median and interquartile range, with whiskers representing the minimum and maximum values. Individual outliers are displayed. Statistical significance (all *p* > 0.05).

Serum GRIN2A concentrations were numerically higher in the gastric cancer group than in the control group (median difference = 78.52 ng/L, 95% CI: −91.11 to 213.33; r = 0.10). In contrast, serum P2RX1 and PD-L1 concentrations were numerically lower in the gastric cancer group, with median differences of 1.16 ng/mL (95% CI: −1.70 to 3.73; r = 0.09) and 42.59 ng/L (95% CI: −67.41 to 158.52; r = 0.09), respectively. TAS and TOS levels also showed no meaningful between-group differences, with negligible effect sizes (r = 0.03 and r = 0.04, respectively). None of the comparisons reached statistical significance, and all confidence intervals for the Hodges–Lehmann differences included zero (all *p* > 0.05) (Table 3, Figure 2).

### 3.4. Assessment of TAS and TOS Across Study Cohorts

TAS and TOS levels were compared between the gastric cancer and control groups. The median TAS level was 0.89 mmol Trolox Eq/L in the gastric cancer group and 0.83 mmol Trolox Eq/L in the control group, with no statistically significant difference between the groups (*p > 0.05*). Similarly, the median TOS level was 16.43 µmol H_2_O_2_ Eq/L in the gastric cancer group and 16.10 µmol H_2_O_2_ Eq/L in the control group, and this difference was also not statistically significant (*p > 0.05*) (Table 3, Figure 2).

**Figure 3:**
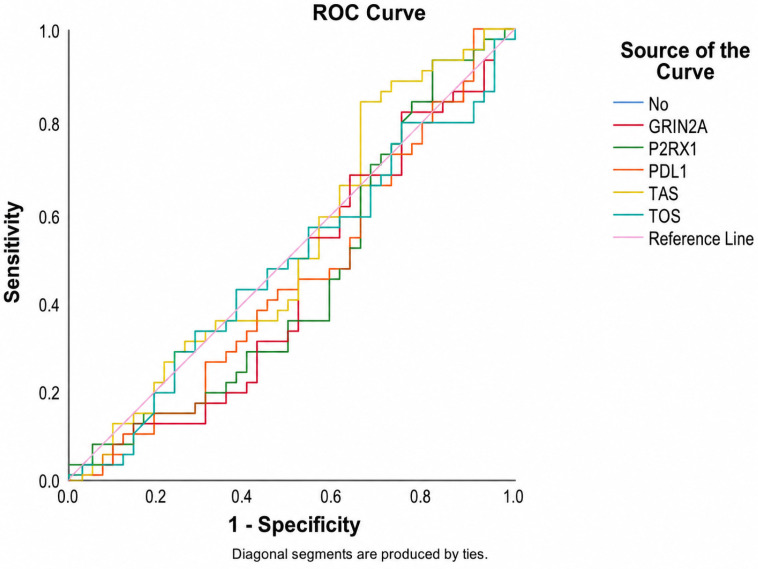
ROC curve graph of ELISA-based GRIN2A, P2RX1, and PD-L1 protein levels together with TAS and TOS parameters in gastric cancer.

Receiver operating characteristic (ROC) analysis showed that the area under the curve (AUC) values for GRIN2A, P2RX1, PD-L1, TAS, and TOS were close to 0.5. The AUC values were 0.559 for GRIN2A, 0.553 for P2RX1, 0.550 for PD-L1, 0.483 for TAS, and 0.521 for TOS. None of the evaluated parameters demonstrated statistically significant discriminatory ability between the gastric cancer and control groups (*p > 0.05* for all). Overall, the ROC analysis indicated that the examined proteins and oxidative stress parameters did not provide meaningful diagnostic performance between the study groups (Table 4, Figure 3).

A multivariable binary logistic regression analysis was performed by entering serum GRIN2A, P2RX1, PD-L1, TAS, and TOS levels simultaneously into the model. The overall model was not statistically significant (Omnibus test: χ^2^ = 2.050, df = 5, *p* = 0.842). The model showed minimal explanatory performance (Cox and Snell R^2^ = 0.023; Nagelkerke R^2^ = 0.030) and poor classification performance, with an overall accuracy of 48.9%. None of the evaluated biomarkers was independently associated with gastric cancer status (all *p* > 0.05) (Table 5). The Hosmer–Lemeshow test was non-significant (χ^2^ = 8.258, *p* = 0.409), indicating no evidence of substantial lack of fit; however, the overall predictive performance of the model remained poor.

Spearman correlation analysis was performed in the overall study population (Table 6). Serum GRIN2A levels showed significant positive correlations with serum P2RX1 (r = 0.511, *p* < 0.001) and PD-L1 levels (r = 0.703, *p* < 0.001). Serum P2RX1 and PD-L1 levels were also positively correlated (r = 0.557, *p* < 0.001). In addition, PD-L1 Ct values showed a positive correlation with TAS levels (r = 0.304, *p* = 0.004). In subgroup analyses, gender was negatively correlated with TOS in the gastric cancer group (r = −0.299, *p* = 0.046), indicating relatively higher TOS levels among female patients. In the healthy control group, gender was positively correlated with TAS (r = 0.352, *p* = 0.018), indicating relatively higher TAS levels among male controls. No other statistically significant correlations were observed.

### 3.5. Kaplan–Meier Survival Analysis

**Figure 4:**
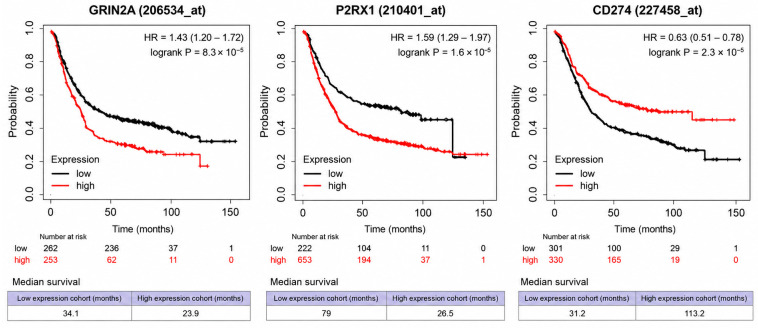
Kaplan–Meier overall survival curves of gastric cancer patients according to *GRIN2A*, *P2RX1*, and *CD274 (PD-L1)* expression levels based on the TCGA gastric cancer dataset. High *GRIN2A* and *P2RX1* expression were associated with significantly poorer overall survival, whereas high *CD274 (PD-L1)* expression was associated with significantly improved overall survival. Hazard ratios (HRs), 95% confidence intervals (CIs), log-rank *p* values, and median survival times are indicated in each panel.

Kaplan–Meier survival analysis based on the TCGA gastric cancer cohort demonstrated significant associations between the expression levels of the investigated genes and overall survival (Figure 4). Patients with high *GRIN2A* expression exhibited significantly shorter overall survival than those with low *GRIN2A* expression (HR = 1.43, 95% CI: 1.20–1.72, log-rank *p* = 8.3 × 10^−5^), with median survival times of 23.9 and 34.1 months, respectively. Similarly, high *P2RX1* expression was associated with poorer overall survival (HR = 1.59, 95% CI: 1.29–1.97, log-rank *p* = 1.6 × 10^−5^), with median survival times of 26.5 months for the high-expression group and 79.0 months for the low-expression group. In contrast, high *CD274 (PD-L1)* expression was associated with significantly better overall survival (HR = 0.63, 95% CI: 0.51–0.78, log-rank *p* = 2.3 × 10^−5^), with a median survival of 113.2 months compared with 31.2 months in patients with low *CD274* expression.

## 4. Discussion

Gastric cancer is a highly heterogeneous malignancy characterized by complex interactions among tumor cells, inflammatory mediators, immune checkpoint pathways, oxidative stress, and cellular signaling networks. These biological processes collectively contribute to tumor development and progression. Increasing evidence suggests that systemic molecular alterations detectable in peripheral blood may partially reflect tumor-associated immune dysregulation and inflammatory responses during gastric carcinogenesis [4]. In the present study, the gene and protein expression levels of GRIN2A, P2RX1, and PD-L1, together with systemic oxidative stress parameters TAS and TOS, were investigated in patients with gastric cancer and compared with those of healthy controls.

RT-PCR analysis showed a numerically higher relative fold change for *P2RX1* in the gastric cancer group, whereas *GRIN2A* and *PD-L1* showed numerically lower fold-change values compared with the control group. However, none of these differences reached statistical significance. Therefore, the present findings do not provide evidence of differential peripheral blood expression or activation of glutamatergic, purinergic, or immune checkpoint-related pathways in gastric cancer. Nevertheless, previous studies have suggested that purinergic receptors and glutamate-associated signaling pathways may contribute to tumor proliferation, apoptosis resistance, metabolic adaptation, and modulation of the tumor microenvironment in different cancer settings [6,7]. These literature-based mechanisms should be interpreted independently of the non-significant findings observed in the present cohort.

GRIN2A, a subunit of the N-methyl-D-aspartate (NMDA) receptor, has been implicated in several malignancies through its role in calcium-dependent signaling pathways, mitochondrial regulation, oxidative stress response, and cellular survival mechanisms. Previous studies have demonstrated that dysregulated NMDA receptor signaling may contribute to tumor progression and resistance to apoptosis in different cancer types, including melanoma and colorectal cancer [6,14]. Although *GRIN2A* has been implicated in several malignancies, the present study did not demonstrate a statistically significant difference in peripheral blood *GRIN2A* expression or serum concentration between the groups. Therefore, no conclusion can be drawn regarding the role of GRIN2A-related glutamatergic signaling in gastric cancer based on the current data. Further studies including tumor tissue analyses and clinically characterized patient cohorts are needed.

Purinergic signaling has emerged as an important regulator of extracellular ATP-mediated communication in the tumor microenvironment. In particular, ATP-dependent purinergic receptor activation has been associated with inflammatory signaling, immune cell recruitment, platelet activation, and modulation of tumor-associated immune responses [7]. Although P2RX7 has been extensively investigated in cancer biology, recent evidence suggests that other purinergic receptors, including P2RX1, may also contribute to inflammatory signaling and systemic immune regulation in cancer [8,12]. In the present study, *P2RX1* expression showed a numerically higher fold-change value in the gastric cancer group; however, this difference was not statistically significant. Accordingly, the present findings do not provide evidence for altered purinergic signaling or systemic immune activation in gastric cancer. This observation may be considered hypothesis-generating and should be examined in larger studies.

*PD-L1* expression has been extensively studied in gastric cancer, particularly in relation to immune checkpoint inhibition and tumor-immune evasion mechanisms. Increased *PD-L1* expression is associated with immune suppression, aggressive tumor behavior, and therapeutic responsiveness in subsets of patients with gastric cancer [9]. In the present study, *PD-L1* gene expression and serum protein levels did not differ significantly between patients with gastric cancer and healthy controls. Although PD-L1 is clinically relevant in gastric cancer tissue and in the context of immunotherapy, the present peripheral blood findings do not support its use as an independent circulating biomarker in this cohort. Differences in tumor stage, molecular subtype, treatment exposure, and the use of peripheral blood rather than tumor tissue may have contributed to the absence of significant findings.

Nevertheless, recent studies suggest that circulating PD-L1 expression profiles detected in the peripheral blood may provide clinically relevant information regarding systemic immune activation and therapeutic responsiveness in gastrointestinal malignancies [10,15,16,17]. The potential value of peripheral blood-based *PD-L1* assessment should be investigated in larger, clinically stratified cohorts.

At the protein level, serum GRIN2A concentration was numerically higher in the gastric cancer group, whereas serum P2RX1 and PD-L1 concentrations were numerically lower. However, none of these differences reached statistical significance. Therefore, the present serum findings do not support a consistent circulating protein signature associated with gastric cancer. Gene expression and protein measurements were obtained from different biological compartments, namely peripheral whole blood and serum; therefore, direct concordance between mRNA and protein levels should not be expected. Similar discrepancies between tissue-level expression and circulating protein levels have been reported for immune-related and inflammation-associated biomarkers in cancer biology [4]. In the present study, the non-significant findings may have been influenced by the biological heterogeneity of gastric cancer, variability in tumor burden and immune status, and the limited ability of circulating biomarkers to reflect localized molecular alterations within the tumor microenvironment [18]. However, these potential explanations remain speculative and should be evaluated in larger clinically characterized cohorts. An important consideration in the interpretation of the present findings is that gene expression and protein measurements were obtained from different biological compartments. While mRNA expression was quantified from peripheral whole blood, circulating protein concentrations were measured in serum. Whole blood RNA primarily reflects transcriptional activity in circulating blood cells, whereas serum protein levels represent proteins released from multiple cellular and tissue sources, including immune cells, damaged tissues, extracellular vesicles, and soluble circulating molecules. Therefore, direct concordance between mRNA and protein levels should not necessarily be expected. Instead, these complementary analyses provide different aspects of the systemic biological alterations associated with gastric cancer.

Oxidative stress has long been implicated in gastric carcinogenesis through mechanisms involving DNA damage, chronic inflammation, mitochondrial dysfunction and dysregulation of intracellular signaling pathways [11]. However, no statistically significant differences in TAS or TOS levels were observed between patients with gastric cancer and healthy controls in the present study. These findings are consistent with those of previous studies, indicating that systemic oxidative stress parameters may not always adequately differentiate cancer patients from controls, particularly in heterogeneous or early stage disease populations [12]. Oxidative stress in gastric cancer may be influenced by multiple factors, including tumor stage, inflammatory activity, nutritional status, metabolic alterations, and treatment-related changes. Therefore, circulating TAS and TOS measurements may not fully represent the localized oxidative alterations occurring within tumor tissues.

ROC analysis further supported the limited diagnostic performance of these biomarkers. The AUC values for GRIN2A, P2RX1, PD-L1, TAS, and TOS were close to 0.5, indicating poor discriminatory performance between gastric cancer patients and healthy controls. Although relatively high sensitivity and specificity values were observed at the selected cut-off points, the absence of statistically significant AUC values suggests that these biomarkers alone are insufficient for standalone diagnostic use [13]. Nevertheless, these findings support current evidence suggesting that single circulating biomarkers rarely achieve sufficient diagnostic accuracy in complex malignancies such as gastric cancer and may instead provide greater clinical value when integrated into multi-marker molecular panels combined with clinicopathological parameters [19,20]. An important consideration when interpreting the present findings is the use of peripheral blood rather than tumor tissue samples for analysis. Although tumor tissue analyses would provide more direct information regarding local molecular alterations within the tumor microenvironment, peripheral blood biomarkers were specifically selected because of their minimally invasive nature, ease of repeated sampling, and potential applicability in longitudinal disease monitoring [21,22]. Therefore, the lack of statistically significant differences observed in the current study may partly reflect the limited ability of circulating biomarkers to fully capture the localized tumor-specific molecular alterations occurring within gastric cancer tissues.

Collectively, the observed numerical increases in GRIN2A, P2RX1, and PD-L1 expression may indicate the presence of systemic molecular and immunological alterations associated with gastric cancer progression. These signaling pathways may interact through inflammatory signaling, oxidative stress responses, calcium homeostasis, and immune checkpoint activation, thereby contributing to tumor-associated immune modulation and disease biology.

## 5. Conclusions

In this study, peripheral blood GRIN2A, P2RX1, and PD-L1 gene expression levels, serum protein concentrations, and oxidative stress parameters did not differ significantly between patients with gastric cancer and healthy controls. In addition, ROC and logistic regression analyses showed that none of the evaluated parameters had meaningful diagnostic or independent predictive performance in the present cohort. Therefore, these markers cannot be considered clinically useful standalone circulating biomarkers for gastric cancer based on the current findings. The results should be interpreted as preliminary and exploratory, particularly given the limited sample size and the absence of detailed clinicopathological stratification. Larger prospective studies including tumor stage, histological subtype, metastatic status, treatment history, and paired tumor tissue–blood analyses are needed to clarify whether these markers may have relevance in specific gastric cancer subgroups or as components of broader biomarker panels.

## 6. Limitations

Several limitations of this study should be considered. First, the relatively limited sample size may have reduced the ability to detect modest between-group differences. Second, gene expression and protein measurements were obtained from peripheral blood and serum rather than tumor tissue; therefore, the findings may not fully represent localized molecular changes within the gastric tumor microenvironment. Although circulating biomarkers are minimally invasive and may be suitable for repeated sampling, their relationship with tissue-level molecular alterations remains uncertain. Third, detailed clinicopathological data, including tumor stage, histopathological subtype, metastatic status, lymph-node involvement, and treatment history, were not available for all patients. Consequently, reliable stratified analyses according to these characteristics could not be performed. This is particularly important because gastric cancer is biologically heterogeneous, and circulating molecular and oxidative stress parameters may vary according to disease stage, tumor burden, subtype, and treatment exposure In addition, functional analyses of receptor-related signaling, inflammatory mediators, and downstream molecular interactions were not performed. Although a multivariable logistic regression model was performed, the limited sample size and the low explanatory and classification performance of the model precluded the development of a robust biomarker prediction model. In addition, longitudinal follow-up analyses were not performed. Therefore, the present findings should be interpreted as preliminary and exploratory. Larger prospective studies incorporating comprehensive clinicopathological characterization, paired tumor tissue and peripheral blood analyses, and appropriately powered subgroup analyses are needed to determine whether GRIN2A, P2RX1, PD-L1, TAS, and TOS have relevance in specific gastric cancer subgroups.

In addition, gene expression normalization was performed using a single reference gene *GAPDH*. Although *GAPDH* is a widely accepted housekeeping gene, the inclusion of multiple validated reference genes would further improve the robustness of RT-qPCR normalization in future studies.

## Figures and Tables

**Table 1:** Comparison of demographic characteristics between gastric cancer and control groups.

Variable	Gastric Cancer (n = 45)	Healthy Controls (n = 45)	Effect Estimate (95% CI)	*p*-Value
Female, n (%)	11 (24.4)	12 (26.7)	OR = 0.89 (0.35–2.30)	*0.809* ^a^
Male, n (%)	34 (75.6)	33 (73.3)	Reference	
Age, years, mean ± SD	63.93 ± 9.91	65.64 ± 10.89	Mean 95% CI: 61.04–66.62 vs. 62.25–68.82 ^b^; Hedges’ g = −0.16 (−0.57 to 0.25)	*0.438* ^c^
Age range, years	40–84	45–87	—	—

Data are presented as n (%) or mean ± SD. OR, odds ratio; CI, confidence interval. ^a^ Chi-square test. ^b^ Bootstrap 95% confidence intervals for mean age based on 1000 samples. ^c^ Mann–Whitney U test. Hedges’ g was calculated for age as a standardized effect size. Statistical significance was set at *p* < 0.05.

**Table 2:** Δ_Ct_, ΔΔ_Ct_, and fold-change analysis of target gene expression.

Gene	Control Δ_Ct_ (Mean ± SD)	Gastric Cancer Δ_Ct_ (Mean ± SD)	ΔΔ_Ct_	Fold Change (2^−ΔΔCt^)	Fold Regulation	*p*-Value
*P2RX1*	2.32 ± 3.45	1.19 ± 4.60	−1.13	2.19	2.19	*0.281*
*PD-L1*	0.64 ± 3.44	1.48 ± 3.97	0.84	0.56	−1.80	*0.658*
*GRIN2A*	−0.24 ± 5.08	0.96 ± 4.57	1.20	0.43	−2.30	*0.579*

ΔCt = Ct (target gene) − Ct(GAPDH). ΔΔCt = ΔCt (gastric cancer group) − ΔCt (control group). Fold change was calculated using the 2^−ΔΔCt^ method. *p* < 0.05 was considered statistically significant.

**Table 3:** Group-wise comparison of protein expression and oxidative status parameters in GC and healthy subjects.

Parameter	Group	Mean ± SD	Min	Max	Median (50th)	Hodges–Lehmann Difference, Case − Control (95% CI)	*p*-Value	Effect Size, r
GRIN2A (ng/L)	Case	1291.24 ± 735.52	108.52	3818.89	1044.81	78.52 (−91.11 to 213.33)	*0.339* ^a^	0.10
	Control	1230.56 ± 703.25	581.48	3623.70	928.89			
P2RX1 (ng/mL)	Case	22.06 ± 9.22	2.53	55.86	20.80	1.16 (−1.70 to 3.73)	*0.388* ^a^	0.09
	Control	22.83 ± 12.10	12.11	72.63	18.21			
PD-L1 (ng/L)	Case	1085.56 ± 572.98	47.78	2410.37	909.63	42.59 (−67.41 to 158.52)	*0.413* ^a^	0.09
	Control	1101.04 ± 721.23	56.30	3580.00	860.00			
TAS (mmol Trolox Eq/L)	Case	1.05 ± 0.69	0.09	3.44	0.89	−0.03 (−0.30 to 0.19)	*0.781* ^a^	0.03
	Control	1.23 ± 1.00	0.07	4.04	0.83			
TOS (µmol H_2_O_2_ Eq/L)	Case	20.31 ± 11.78	4.72	44.32	16.43	0.71 (−3.63 to 4.85)	*0.732* ^a^	0.04
	Control	19.32 ± 11.11	5.27	43.96	16.10			

Data are presented as mean ± SD, minimum–maximum, and median values. ^a^ Mann–Whitney U test. TAS, total antioxidant status; TOS, total oxidant status. Hodges–Lehmann differences are presented as Case − Control with 95% confidence intervals. Effect size was calculated as r = |Z|/√N (N = 90). Statistical significance was set at *p* < 0.05.

**Table 4:** ROC analysis results of ELISA-derived GRIN2A, P2RX1, and PD-L1 protein levels and oxidative stress parameters (TAS and TOS).

Parameter	GRIN2A	P2RX1	PD-L1	TAS	TOS
AUC	0.559	0.553	0.550	0.483	0.521
95% CI	0.438–0.679	0.431–0.674	0.430–0.670	0.362–0.604	0.401–0.641
*p* value	*0.339*	*0.388*	*0.413*	*0.781*	*0.732*
Cut-off	896.85	19.32	909.44	0.83	34.46
Sensitivity (%)	77.8	62.2	51.1	62.2	20.0
Specificity (%)	42.2	60.0	64.4	48.9	91.1

AUC, area under the curve; CI, confidence interval; TAS, total antioxidant status; TOS, total oxidant status. Significant indicates *p* < 0.05.

**Table 5:** Multivariable binary logistic regression analysis of serum biomarkers associated with GC status.

Variable	B	S.E.	*p* Value	Exp(B)	95% CI (Lower–Upper)
GRIN2A	0.000	0.000	0.427	1.000	0.999–1.001
P2RX1	0.010	0.027	0.706	1.010	0.959–1.064
PDL1	0.000	0.001	0.634	1.000	0.999–1.001
TAS	0.263	0.259	0.309	1.301	0.784–2.160
TOS	−0.010	0.019	0.592	0.990	0.953–1.028

None of the variables were independently associated with GC (*p* > 0.05). B: regression coefficient; S.E.: standard error; Exp(B): odds ratio; CI: confidence interval.

**Table 6:** Spearman Correlation Analysis Among Demographic, Serum Biomarker, Gene Expression, and Oxidative Stress Parameters.

Analysis Group	Variable Pair	Spearman’s *r*	*p*-Value
Overall population	GRIN2A–P2RX1	0.511	*<0.001* *
Overall population	GRIN2A–PD-L1	0.703	*<0.001* *
Overall population	P2RX1–PD-L1	0.557	*<0.001* *
Overall population	PD-L1 Ct–TAS	0.304	*0.004* *
GC group	Gender–TOS (Higher TOS in females)	−0.299	*0.046* *
Healthy control group	Gender–TAS (Higher TAS in males)	0.352	*0.018* *

* Statistically significant at *p* < 0.05.

## Data Availability

The original contributions presented in this study are included in the article. Further inquiries can be directed to the corresponding author.

## Abbreviations

The following abbreviations are used in this manuscript:

AUC	Area under the curve
CD274	CD274 Antigen
Ct	Cycle threshold
ELISA	Enzyme-linked immunosorbent assay
GAPDH	Glyceraldehyde-3-phosphate dehydrogenase
GRIN2A	Glutamate receptor ionotropic N-methyl-D-aspartate 2A
NMDA	N-methyl-D-aspartate
PD-L1	Programmed death-ligand 1
P2RX1	Purinergic receptor P2X1
ROC	Receiver operating characteristic
RT-PCR	Real-time polymerase chain reaction
TAS	Total antioxidant status
TOS	Total oxidant status
